# The day-of-the-week effect is resilient to routine change

**DOI:** 10.3758/s13421-024-01606-8

**Published:** 2024-07-16

**Authors:** Anna M. A. Wagelmans, Virginie van Wassenhove

**Affiliations:** CEA/DRF/Joliot, NeuroSpin - INSERM Cognitive Neuroimaging Unit, U992, Université Paris-Saclay, Bat 145 PC 156, F-91191 Gif-sur-Yvette, France

**Keywords:** Temporal cognitive map, Chronometry, Week, COVID-19, Stringency

## Abstract

Temporal landmarks are salient events that structure the way humans think about time. They may be personal events, such as one’s birthday, or shared cultural events, such as the COVID-19 pandemic. Due to societal habits, the cyclical weekly structure – for example, working on weekdays, resting on the weekends – helps individuals orient themselves in time. In the “day-of-the-week effect,” individuals are faster at reporting which day of the week it is on weekends than they are on weekdays. Herein, we hypothesized that the disruption of social habits during the COVID-19 pandemic lockdowns may have weakened this effect, thereby accounting for the “Blursday” phenomenon. In the current study, speeded responses to the question “What day of the week is it?” were collected online from 1,742 French participants, during and after the lockdown periods. We found that reaction times for days of the weekends remained faster than for weekdays during the lockdown, although the overall reaction times were significantly slower during lockdown. We also found that responses were slower as governmental stringency rules and restrictions in mobility increased. Our results suggest that the weekend landmark remains a stable temporal anchor in French culture despite the experienced temporal distortions induced by the disruption of social habits during the pandemic. We conclude that cultural temporal landmarks shape socially shared temporal cognitive maps.

**Supplementary Information:**

The online version contains supplementary material available at 10.3758/s13421-024-01606-8.

## Introduction

By analogy to spatial landmarks, temporal landmarks are salient events that structure the way we mentally navigate time. Important personal events, such as one’s birthday, tend to be used as landmarks in autobiographical memory (Shum, 1998). Recurring landmarks such as weekends also tend to structure the way we think about time. Although we may commonly have a linear representation of time and of events in time, weeks have a circular pattern: they reoccur at a fixed frequency. As such, they can define temporal distances by providing a (social and cultural) metric for time. Weekends introduce a hierarchical organization within the week, which allows anchoring one’s present position in time, but also structures memory and planning. Indeed, it has been shown that individuals more easily remember an event that happened during a weekend than during any other day of the week (Friedman, 1987; Huttenlocher et al., 1992). When deciding to start a new activity, we tend to plan it for the beginning of the week, or another landmark such as the beginning of the month or the year (Dai et al., 2015; Dai & Li, 2019; Hennecke & Converse, 2017). Both personal and socially defined temporal landmarks structure the way we think about the past and about the future. In experimental psychology, such landmarks can be investigated through chronometry (reaction times (RTs)) and performance (error rate (ER)) in tasks where one must locate a point in time.

In their original study, Koriat and Fischhoff (1974) showed that participants' RTs and ERs to the question “What day of the week is it?” varied as a function of the distance of the day to the weekend, such that RTs were slower and ERs higher the further away the day was from the weekend. This suggested that the weekend is used as a landmark when orienting in time. The experiment, originally conducted in Israel, was replicated in the USA and in France (Carreras et al., 2008; Shanon, 1979), where social habits such as working during the week and resting or doing other activities on the weekend may drive this day-of-the-week (DoW) effect. Importantly, Koriat and Fischhoff (1974) argued that this pattern of RTs does not match a classic serial memory search. If that were the case, participants would be counting off days, starting from the beginning of the week until they reached the target day: RTs would thus increase linearly along the week. The observed pattern suggests a different underlying search mechanism. Instead, the authors proposed a model in which one step consists of a coarse search so that one roughly examines if the day is in the beginning, middle, or end of the week. This search implies that weekends, as temporal landmarks or boundaries, facilitate the search by fastening the process for the beginning and the end of the week – i.e. days closest to the landmark. In this model, the anchoring effect of weekends constitutes a facilitating role by setting boundaries and thus, a temporal metric to the memory search. While other cues, such as personal weekly habits, could facilitate this search, the weekend by virtue of being shared across the population likely drives the overall pattern of RTs.

During the worldwide pandemic, several strict lockdowns interrupted common patterns in the social structuring of the week, possibly disrupting the anchoring effect of the weekend on the postulated cognitive temporal map of the week cycle. For instance, one study (Fedrigo et al., 2023) investigated the relationship between the perceived salience of the week cycle and the weekly variations in risk tolerance during the pandemic. The authors tested participants during the two UK lockdowns, and reported a decline in risk tolerance from Monday to Thursday followed by an increase to risk tolerance on Friday. This risk tolerance pattern was further modulated by participants’ sense of the weekday. In contrast, later in the pandemic (November 2020), risk tolerance did not show weekly variations, even among participants with a strong sense of “weekday.” When considered together, these results suggest that social reinforcement of the week cycle structure is necessary to preserve it, as it was weakened during the successive pandemic lockdowns. Aside from the many observations that participants’ sense of time was distorted during the lockdowns (Chaumon et al., 2022; Cravo et al., 2022; Droit-Volet et al., 2020; Ogden, 2020, 2021), a few studies also reported temporal disorientations, which could be associated with possible perturbations of temporal landmarks. For instance, a measure of temporal self-location was affected during the pandemic (Velasco, Perroy, et al., 2022b), the temporal distances to self were distorted during lockdown (Chaumon et al., 2022), errors in the placement of events in one’s mental time-line increased during the pandemic (Pawlak & Sahraie, 2023), and, ultimately, the retrospective assessment of lived temporalities during the pandemic showed that the distance between memorable events during the lockdown were closer than those outside of it (Rouhani et al., 2023). Altogether, the empirical observations gathered during the pandemic period suggest a perturbation of the temporal landmarks that may typically ground our sense of time on a daily basis.

Thus, if temporal landmarks are flexibly grounded in cultural habits, we hypothesized that their disruption during the COVID-19 pandemic would induce slower RTs or a disrupted pattern of RTs for the week days (e.g., Fedrigo et al., 2023), and an increased rate of errors to the question “What day of the week is today?” The present study was conducted at different moments during the pandemic, and included behavioral data collected while participants were under lockdown and outside of it. We prompted participants of the Blursday study (Chaumon et al., 2022) unexpectedly, at various times over days of testing, with the question “What day of the week is it?”

To assess the impact of lockdown, we used a stringency and a mobility index (Google LLC, 2021; Hale et al., 2021; Mathieu et al., 2020): the stringency index reflects how stringent the government measures were throughout the pandemic, as assessed by public gathering and traveling restrictions, school and workplace closures, and stay-at-home requirements; the mobility index quantifies how much time visitors spent in subway stations, rental stations or taxi stands. Taken together, stringency and mobility indices can be used as proxies of the degree to which participants’ daily life was disrupted and mobility changed, respectively. We selected stringency and transit mobility indices as measures of the disruption in social habits induced by the COVID-19 pandemic because they provide standardized metrics for governmental responses: the stringency index was computed as part of the Oxford COVID-19 Government Response Tracker project (Hale et al., 2021), whereas the transit mobility index was part of Google’s COVID-19 Community Mobility Reports and derived from anonymized data provided by Google apps (Google LLC, 2021). Both indexes have been used in research in health policy (Hadjidemetriou et al., 2020; Hale et al., 2020; Islam et al., 2020; Koh et al., 2020; Noland, 2021; Tan-Torres Edejer et al., 2020; Wellenius et al., 2021), environment policy (Liu et al., 2021; Venter et al., 2020), politics (Chen et al., 2021, 2023), economy (Akter, 2020; Bonadio et al., 2021; Gottlieb et al., 2020), and urban planning (Hasselwander et al., 2021; Thombre & Agarwal, 2021). In particular, the mobility index has been shown to reliably predict COVID-19 case incidence (Sulyok & Walker, 2020). The Blursday database provides a comprehensive description of how both stringency and mobility correlated with behavioral changes in different temporal tasks (Chaumon et al., 2022). For example, stringency and mobility indices both correlated with the retrospective estimation of duration: participants underestimated durations with increasing stringency, but overestimated durations with decreasing mobility.

We predicted that RTs would slow down with increasing levels of stringency and decreasing levels of transit mobility. Additionally, we hypothesized that if the anchoring role of the weekends was disrupted during lockdown, the effect of stringency and transit mobility would be more prominent around the weekend than in the middle of the week.

## Methods

### Participants

**Fig. 1:**
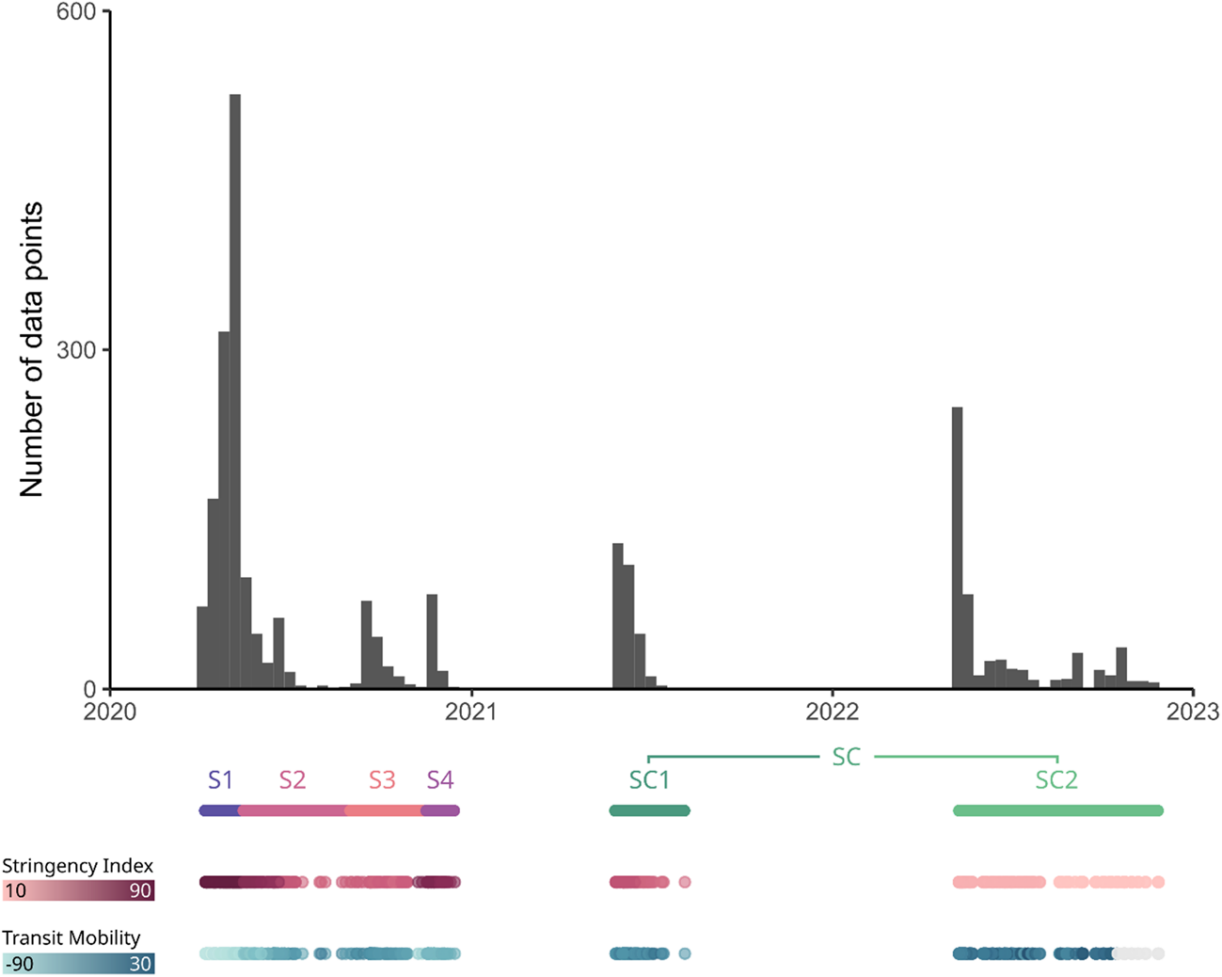
Chronology of data collection across sessions. Data were collected across four lockdown sessions of varying stringency (S1, S2, S3, and S4) between April 2020 and December 2020, and two control sessions collected in May 2021 (SC1) and again in November 2022 (SC2). The first line of colored bars delineates the four sessions (S1 is purple, S2 is dark pink, S3 is pink, S4 is mauve, SC1 is green, SC2 is light green). The second line of red colored dots shows the intensity of stringency over time (darker red being more stringent), as estimated by the stringency index. The third line of blue colored dots shows the changes in transit mobility (darker blue representing higher mobility), which were estimated by the transit mobility index. Gray dots show missing data; after 15 October 2022 the transit mobility index was no longer recorded by Google

A total of 2,488 responses (from 1,742 participants) were collected in this study over multiple sessions spanning from April 2020 to November 2022 (Fig. 1). 1,911 data points from 1,165 participants were collected from the French Temporal Landmarks task of the Blursday database (Chaumon et al., 2022): 1,176 data points (from 920 participants) were collected during the first lockdown from April 2020 to May 2020 (Blursday Session 1; S1) and 100 data points (from 100 participants) during the second lockdown from November 2020 to December 2020 (Blursday Session 4; S4). The first lockdown was much stricter than the second one; for example, schools were not closed during the second lockdown. Two sessions of data collection took place in between the lockdowns. First, 170 samples (from 106 participants) were collected in May 2020, about 2 weeks after the first lockdown (Blursday Session 2; S2). Then, 160 samples (from 76 participants) were collected in August 2020, 3 months after the first lockdown (Blursday Session 3; S3). These two sessions correspond to intermediary periods during which government measures still restricted daily life but no lockdowns were in place. About a year after the first lockdown, in May 2021, 303 samples (180 participants) were collected from a new set of participants, and were completed in 2022 by an additional 577 samples (from 577 participants) to increase the otherwise low statistical power of the original data collection, as assessed by post hoc power analyses. These two datasets were defined a priori as control conditions (SC1 and SC2), corresponding to periods of low stringency, and were pooled together for our analysis. As such, the control session (SC) totalizes 880 samples (from 757 participants), collected from May 2021 to November 2022.

The 577 additional participants in SC2 were recruited partly through Prolific (www.prolific.co), and data have been added to the Blursday database (Chaumon et al., 2022). Otherwise, participants in the Blursday database were recruited by means of general advertisement using institutional newsletters such as the RISC (Relais d’information sur les sciences cognitives, www.risc.cnrs.fr), and/or outside the institution through social media, namely, Twitter / X (www.twitter.com) and Facebook (www.facebook.com) posts relayed by the researchers of the consortium. Subscribers to the newsletters or followers of these accounts received announcements describing the scope of the study and the link to participate. They were also provided with an email dedicated to the study where they could send any concerns or questions directly to the P.I. of the study. Participants in SC1 were given an option to receive a small compensation for their participation and ~44% of them chose so (80 out of 180 participants). Participants in SC2 were recruited on Prolific and were paid through their Prolific account, in accordance with the platform’s policies. Pay was set at £0.40 for a 1- to 3-min long study, amounting to an average hourly rate at £21.5. No compensation was given in the other sessions.

At the beginning of each session, participants self-reported whether they had consumed drugs (specifically, illicit substances such as marijuana) in the 24 h preceding the experiment, and whether they had previously been given a neurological or psychiatric diagnosis. When participants answered positively to any one of these questions, they were a priori excluded from data collection and logged out of the site.

### Data acquisition procedure

Data acquisition in the study was realized through Gorilla Experiment Builder (Anwyl-Irvine et al., 2020). The French Temporal Landmarks task is available at: https://app.gorilla.sc/openmaterials/278096.

### Protocol

The full experimental protocol (detailed elsewhere for all countries, see Chaumon et al., 2022) consisted, in France, of four longitudinal sessions (S1, S2, S3, S4; same participants) and two control sessions grouped as one (SC; new participants tested after the pandemic, in 2021 and in 2022; see Fig. 1).

In each session, participants had to answer a series of questionnaires and tasks provided to them in pseudo-random order. Each session took a few hours to complete. Participants did not complete the full session at once, and logged in several times and over several days to complete the different tasks. The temporal landmarks task consisted of asking participants to respond as fast as possible to the following two successive questions: “What day of the week is it?” and “What is an important event for you today?” Herein, we solely focus on the question “What day of the week is it?”

Participants answered this question up to three times in S1, S2, and S3, up to two times in SC, and only once in S4. Participants’ reaction times (RTs) were collected on the first key entry in the dedicated answering window.

### Statistical analysis

#### Treatment of responses

Participants’ responses consisted of a text input. These were automatically matched with one of the days of the week, as commonly written in French (e.g. “lundi,” “mardi,” etc.). When a participant’s response could not be automatically matched (86 responses out of 2,488), the response was treated manually. There were three possible cases: first, a participant’s response clearly matched one day of the week but had a typo (43 responses) or was written in English (four responses). In this case, the response was considered valid and corrected. Second, the participant answered by giving the full date, including the day of the week, such as “vendredi 10 avril 2020” (19 responses). In this case, we considered that the participants did not complete the same task, and that their response could not be analyzed for our question of interest; these responses were counted as invalid and excluded from the analysis. Third, responses were not identifiable as a specific day of the week (19 responses) and were excluded from the analysis. In total, 38 invalid responses were excluded, representing 1.52% of total data points.

#### Outliers

To exclude outliers, we first applied an absolute cutoff, based on the task demands, assuming that reading the question and answering it should reasonably take no more than 8 s. We then applied a relative cutoff: we grouped the data by session and excluded the points beyond the 95 central percentiles. These two rules removed 157 data points (6.40% of valid data points). Additionally, to homogenize the data collected through all sessions, we applied an exclusion criterion on the time of day and the time of year. We excluded all data points collected during the night (i.e., from midnight to 6 a.m.) and data points collected in the months of July and in August, as this summer period is a massively followed French holiday season. Indeed, during these two months, long periods of vacation replace the usual weekly work cycle. Following our hypothesis that social habits ground the DoW effect, the months of July and August would not be comparable to other months. This removed 177 data points (7.72% from the previous step) without impacting the pattern of RTs (Fig. S2, Online Supplemental Material (OSM)). Last, only correct responses were considered for the RT analysis, excluding 62 points (2.93% of cleaned data) from the analysis. Thus, and in total, 2,054 data points from 1,453 participants (female = 878, male = 438, no answer = 137, *M*_*age*_ = 38.6 years, *SD* = 15.4 years; see Table S2 (OSM) for detailed demographics) were used for the analysis, excluding 396 data points (16.16% of valid data points).

#### Stringency and transit mobility indices

The Blursday database collected and formatted the Stringency Index from OurWorldInData (Mathieu et al., 2020). The stringency index is a composite measure of nine governmental response indicators developed as part of the Oxford Coronavirus Government Response Tracker project (Hale et al., 2021) that includes school closures, workplace closures, cancellation of public events, restrictions on public gatherings, closures of public transport, stay-at-home requirements and travel bans. It is rescaled to a value ranging from 0 to 100, with 100 being the strictest stringency, in order to provide a metric to compare governmental responses across countries and time. The Google Transit Station Mobility Index (Google LLC, 2021), also provided in the Blursday database, quantifies how much time visitors spent in various transit stations (subway, taxi stand, rentals) during the selected period relative to a baseline period. In the Google dataset, the baseline was defined as the median value from a 5-week period spanning 3 January to 6 February 2020. Hence, the more negative the mobility index, the less mobility compared to baseline. The use of these two indices served as a proxy to the session, which, although carefully aligned to the governmental lockdowns and state of emergency rules, did not strictly map to the levels of stringency and mobility. These two objective measures were shown to be adequate covariates to explore the effect of lockdown on time perception (Chaumon et al., 2022).

#### Models

All analyses were conducted using R (v4.1.1; R Core Team, 2021) with packages tidyverse (v1.3.1; Wickham et al., 2019), lmerTest (v3.1.3; Kuznetsova et al., 2017), effects (v4.2.0; Fox, 2003; Fox & Weisberg, 2019), emmeans (v1.7.2; Lenth, 2022), and car (v3.0.4; Fox & Weisberg, 2019).

As government responses and their impact on daily life was not homogeneous across sessions, we aimed to test for the DoW effect in two ways. In a first analysis, we examined the contrast between the “lockdown” condition (S1, strictest lockdown) and SC. In a second analysis, we used stringency and transit mobility indices as measures of the impact of government responses on daily life, which allowed us to use all data points from all sessions.

To test the existence of a DoW effect in the control and the lockdown sessions, we performed a linear regression model of log-transformed RT over the DoW, separately for S1 (first lockdown, most stringent) and SC (after the last lockdown took place). The DoW were modeled as an ordered categorical variable, with seven levels and Monday as the first day of the week. Participants were modeled as a random variable (ID). The model in R was defined as: *log(RT) ~ DoW + (1 | ID).* For each estimate, we report the t-value and its p-value, using Satterthwaite's method for degrees of freedom. We also report the 95% confidence interval (CI) of the estimate. Unless stated otherwise, all results are reported on the log scale. Post hoc paired t-tests were performed between pairs of days. We report the t-value and the adjusted p-value associated with it (Tukey correction for multiple comparisons), as well as the 95 % CI of the ratio between each pair of days, on the log scale.

To test for the interaction between lockdown and day of the week, we built a linear regression model following a stepwise procedure. We tested models with the Akaike Information Criterion (AIC). The lockdown was modeled as a binary variable (True for S1 and False for SC). The model with only DoW as predictor and the model with both DoW and lockdown as predictors shared similar AICs (548.34 and 548.45, respectively). The model with DoW, lockdown, and their interactions had the highest AIC (AIC = 582.17, 6.17% increase from the lowest AIC). The interaction model was defined in R as: log(RT) ~ DoW * lockdown + (1 | ID).

Furthermore, to test the effect of stringency and transit mobility on RTs over all sessions, we ran several linear mixed-effect models, incrementing the number of terms following a stepwise procedure using AIC. The stringency index and the transit mobility index were modeled as continuous variables and were scaled for the analysis. For stringency, the model with only DoW as predictor and the model with both DoW and stringency index as predictors had similar AICs (496.6 and 497.73, respectively). The model with both DoW, lockdown, and their interactions showed the highest AIC (AIC = 537.18, 8.17% increase from the lowest AIC). The interaction model was defined in R: log(RT) ~ DoW * stringency index + (1 | ID). For transit mobility, the model with both DoW and transit mobility as predictors, but without interaction, had the lowest AIC (AIC = 440.21). The model with DoW only (AIC = 464.56, 5.53% increase from the lowest AIC) and the model with both DoW, transit mobility, and their interaction (AIC = 479.34, 8.89% increase from the lowest AIC) showed a higher AIC. The interaction model was defined in R as: *log(RT) ~ DoW + transit mobility index + (1 | ID).*

An analysis of deviance using type II Wald chi-square tests was used to test for the significance of each predictor. We report the chi-value, and its p-value.

#### Power analysis

The original recruitment sessions were defined by time constraints, in order to match closely with government lockdowns. As such, we did not control for the number of participants, or the sampling per day of the week. We performed post hoc simulation-based power analysis, using the simr (v1.0.5; Green & MacLeod, 2016) package in R to run Monte Carlo simulations on the models fitted on the available data. We define the power as the proportion of significant simulations over all 1,000 simulations that were run for each model. We report the estimated power, as well as the 95% CI of the estimation.

## Results

### Days-of-the-week effect

**Fig. 2:**
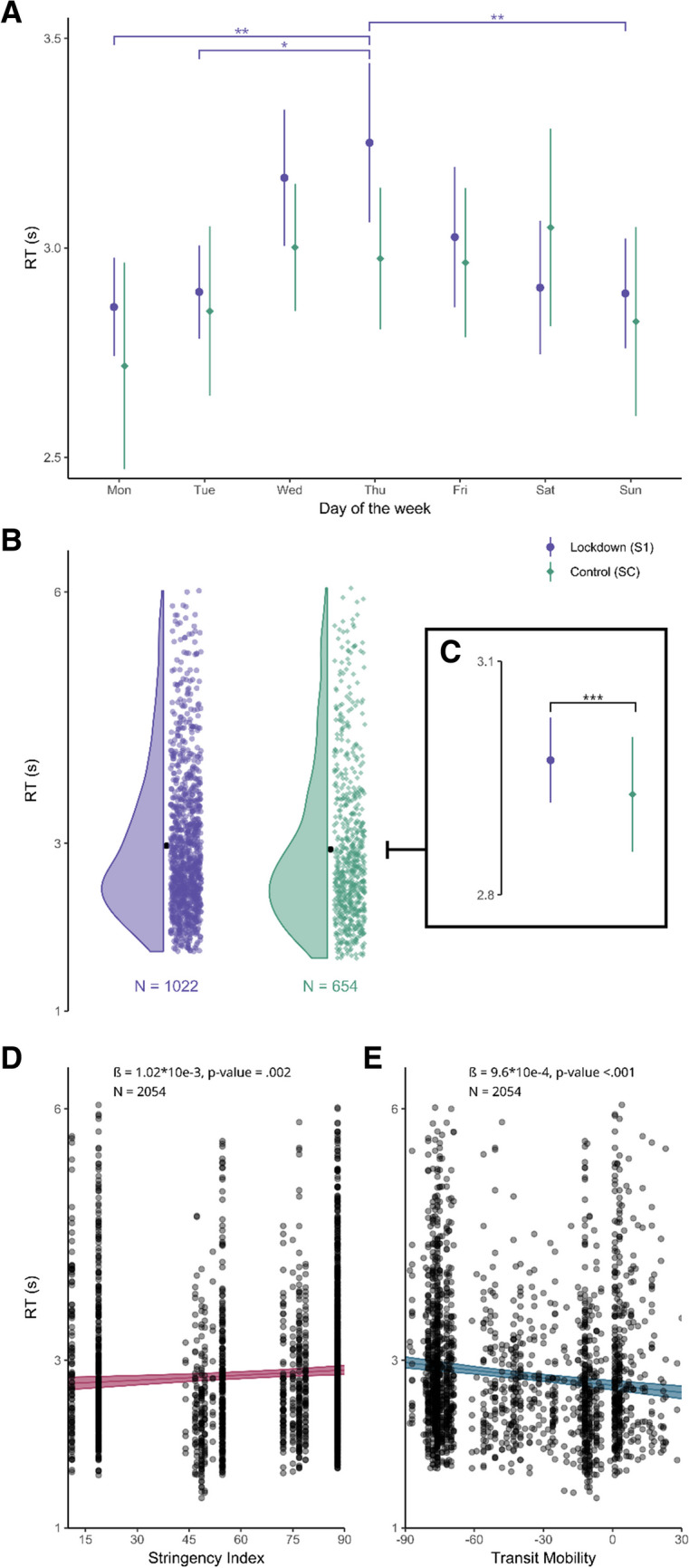
Day-of-the-week effect in and out of lockdown. **A** Reaction times (RTs) in response to the question “What day is it today?” collected each day of the week during lockdown (purple) and during the control session (green). Points are mean estimates. Error bars are two standard errors of the mean (SEM). DoW effect: responses in the middle of the week were slower than at the beginning or at the end of the week. **B** Density plot of RTs in lockdown (purple) and in control (green) session. Black dots are the mean estimates for each session. **C** Insert of B panel. Points show the mean estimate of RTs in each session. Error bars are two SEMs. **D** RTs as a function of stringency and regression line of the model (with two SEMs) showing that RTs significantly increased with increased stringency measures. **E** RTs as a function of transit mobility and regression line of the model (with two SEMs) showing that RTs significantly decreased with increased mobility

The first question we asked was whether we could replicate the DoW effect in data collected online. For this, we performed a linear mixed-effect regression of log-transformed RT over the DoW, separately for S1, where the lockdown was the most stringent, and SC*.* In the control session (654 data points), we found an effect of the DoW, with a significant quadratic trend (*t*(629) = -2.99, *p* = .003, CI = [-0.16, -0.03]) (Fig. 2A). The power analysis yielded a power of 86.60% (CI = [84.33, 88.65]). However, we performed post hoc paired t-tests between pairs of days and found no significant differences between pairs of days. During lockdown (1,022 data points), we also found a DoW effect with a significant quadratic trend (*t*(985) = -4.41, *p* < .001, CI = [-0.14, -0.05]) (Fig. 2A). The power analysis for the quadratic trend yielded a power of 99.50% (CI = [98.84, 99.84]). Post hoc paired t-tests between pairs of days showed a significant contrast between Monday and Thursday (*t*(961) = -3.75, *adjusted p* = .004, CI = [-0.21, -0.02]), Tuesday and Thursday (*t*(977) = -3.57, *adjusted p* = .007, CI = [-014, -0.04]), Thursday and Saturday (*t*(830) = 3.05, *adjusted p* = .038, CI = [0.004, 0.23]), and Thursday and Sunday (*t*(829) = 3.58, *adjusted p* = .007, CI = [0.02, 0.21]). For the DoW pattern in other sessions, see Fig. S1 (OSM).

Despite our best efforts, some participants were prompted several times with the DoW question within one day (up to three times in S1, S2, and S3, up to two times in SC). Koriat and Fischhoff (1974) have shown that participants who answered the DoW question several times during the day were faster and showed a weaker DoW effect than those who answered only once. To check for the robustness of our observations, we conducted additional analyses. First, we qualitatively replicated the original observation and found that first responses were overall slower than the following ones and, most importantly, the DoW effect vanishes for non-first responses (Fig. S3, OSM). Second, we further confirmed that the DoW remained robust when excluding non-first responses during lockdown (*t*(893) = -4.291, *p* < .001, CI = [-0.136, -0.051]) and in the control session (*t*(600) = -3.295, *p* = .001, CI = [-0.171, -0.043]; Fig. S4, OSM). The power analysis yielded a power of 98.80% (CI = [97.91, 99.38]) for the DoW effect in lockdown, and 91.60% (CI = [89.71, 93.24]) for the DoW effect in the control session.

Taken together, a DoW effect could be found in both sessions as captured by a quadratic effect in the model with more robust differences across days seemingly observed during the lockdown.

### Lockdown chronometric effect

To directly test for an effect of lockdown on RTs, we contrasted the data collected during the first lockdown (S1) with those of the control session (SC). In order to test for an effect of both day of the week, lockdown, and their interactions, we built a model including all these terms and ran an ANOVA. We found a significant main effect for the DoW (*X*^*2*^ (6, *N* = 1676) = 31.30, *p* = < .001) and lockdown (*X*^*2*^ (1, *N* = 1676) = 8.39, *p* = .004). However, we found no significant interactions between DoW and lockdown (*X*^*2*^ (6, *N* = 1676) = 5.37, *p* = .50). Therefore, we report the output of the model with both DoW and lockdown, but not their interaction. A significant general slowing down of RTs was found during lockdown compared to the control session (*t*(1370) = 2.9, *p* =.004, CI = [0.01, 0.07]). The same observation was preserved on the first responses-only dataset (*t*(1384) = 2.468, *p* = .014, CI = [0.008, 0.068]; Fig. S4, OSM). The power analysis yielded a power of 70.10% (CI = [67.16, 72.92]). Back-transformed mean estimates (in seconds) collected during lockdown were: M = 2.91, CI = [2.85, 2.96], and outside of lockdown: M = 2.79, CI = [2.72, 2.85] (Fig. 2C). The power analysis for the effect of lockdown yielded a power of 81.50% (CI = [78.95, 83.86]).

### Stringency chronometric effect

We now turn to continuous measures reflecting the degree to which participants lived under stringent conditions. The stringency index is a normalized measure of the stringency of the government responses, allowing us to assess the impact of government responses across all sessions. We asked whether the stringency of governmental measures affected RTs and whether there was an interaction between the DoW and stringency. For this, we ran an ANOVA on the interaction model of DoW and lockdown and found a significant effect for both the DoW (*X*^*2*^ (6, *N* = 2054) = 44.31, *p* = < .001) and the level of stringency (*X*^*2*^ (1, *N* = 2054) = 10.12, *p* =.001). Their interaction was not significant (*X*^*2*^ (16, *N* = 2054) = 9.26, *p* = .16). Therefore, we report the model without the interaction. Results yielded a significant slowing down of RTs with increasing stringency (*t*(1931) = 3.17, *p* = .002, CI = [0.007, 0.032]) (Fig. 2D). The power analysis for the effect of stringency yielded a power of 88.50% (CI = [86.36, 90.41]).

### Transit mobility chronometric effect

Last, we tested whether the effective change in transit mobility induced by governmental measures affected RTs. Similar to the stringency index, this measure allows us to assess the impact of reduced mobility across all sessions. In particular, we aimed to test whether there was an interaction between the DoW and the effect of transit mobility. An ANOVA on the interaction model showed a significant main effect of DoW (*X*^*2*^ (6, *N* = 2011) = 55.33, *p* = < .001) and transit mobility (*X*^*2*^ (1, *N* = 2011) = 35.57, *p* = < .001), but their interaction was not significant (*X*^*2*^ (6, *N* = 2011) = 10.45, *p* = .11). A significant slowing down of RTs was observed with decreasing mobility (*t*(1993) = -5.95, *p* < .001, CI = [-0.05, -0.02]) (Fig. 2E). The power analysis for the effect of transit mobility yielded a power of 100% (CI = [99.63, 100]).

## Discussion

In this study, we replicated the DoW effect, both in and out of lockdown. Interestingly, this effect persisted despite a general slowing down of RTs in lockdown. This implies that the anchoring role of the weekend was maintained during lockdown in a context where common social habits are disrupted to the point of affecting orienting in time (Cellini et al., 2020; Chaumon et al., 2022; Cravo et al., 2022; Droit-Volet et al., 2020; Ogden, 2020, 2021; Van Wassenhove, 2022; Velasco, Gurchani, et al., 2022a, 2022b, 2022c; Velasco, Perroy, et al., 2022b, 2022a) for weeks and months at a time.

During lockdown, Thursday was the day for which RTs were slowest, whereas the days of the weekend were fastest (Fig. 2A). This signifies that the DoW effect was slightly asymmetrical in that RTs increased from Monday to Thursday, and then decreased from Thursday to the weekend days. This pattern contrasts with previous studies in which Wednesday rather than Thursday induced longer RTs (Carreras et al., 2008; Jonas & Huguet, 2008; Koriat & Fischhoff, 1974; Shanon, 1979). This one-day shift might be grounded in differences at the cultural or individual level. Indeed, as shared temporal landmarks seem to be grounded in cultural habits (Dai et al., 2014; Jonas & Huguet, 2008; Koriat & Fischhoff, 1974), an interesting research avenue would be to explore cross-cultural differences of temporal anchors. The anchoring role of the weekend has been found in studies carried out in Israel, where the day of Shabbat would be considered as the most important day of the week, as well as in France or in the USA, where religious cultural heritage instead observes Sunday. All these studies found the peak of the DoW effect to be on Wednesday. However, it would be interesting to test this more widely in cultures where the 2-day weekend does not act as a strong anchor. For instance, in some Arabian countries, which adopted the Friday and Saturday as weekends, or in Iceland, where a 4-workday week was adopted. Beyond the scale of the week, temporal landmarks defined by month and year, have also been reported in France and in the USA (Dai et al., 2014; Dai & Li, 2019; Valax et al., 1996). Inter-cultural investigations would be enriching and help understand whether temporal cognitive maps can be culturally and socially grounded, and how they may shape, in turn, an individual’s behavior. An interesting case would be that of individuals interacting with other cultures who may not share the same temporal landmarks.

On the individual level, this also suggests the need to understand the relation between different groups within one population, and the structure of its cultural environment. Although this study investigated the disrupting effect of a major event like the COVID-19 pandemic, disruptions in one’s life may more commonly affect one’s temporal cognitive map. For example, individuals who were forced to work at home during the pandemic, and returned to working at their workplace afterwards, could have been more affected than individuals who usually work at home. Accordingly, the visual inspection of demographic data suggested that participants who worked at home, whether in lockdown or outside of lockdown, exhibited a DoW effect that peaked on Wednesday. On the other hand, participants who returned to working at their workplace once the lockdown was over showed a peak on Thursday (Fig. S6, OSM). This suggests that work habits, and in particular remote working, may shift the usual weekly cycle. This might also be the case when work habits are atypical, such as night shifts, or working on the weekend (Fig. S9, OSM). For additional information about work habits inside and outside of lockdown, and how these impact the DoW effect, see the OSM. To which extent culturally defined landmarks dominate an individual’s temporal map could be rigorously assessed through an individual’s chronometric pattern according to the day of the week. Such outcomes may provide interesting testable links with social theories of time (Nowotny & Plaice, 2018; Rosa & Trejo-Mathys, 2013), and more specifically the cognitive and neural grounding levels of temporalities (Fraser, 1992; Michon, 1983).

As culturally grounded temporal landmarks facilitate one’s orientation in time, they may be essential in maintaining social and cultural habits, allowing one to live in synchrony with other individuals. However, this ability might be disrupted in some cases. In the case of dementia or psychopathologies known to display temporal disorientations, developing simple mental orientation tests may help early diagnosis and in turn guide remediation (e.g., Peters-Founshtein et al., 2018). Patients suffering from Alzheimer’s disease or clinical depression may benefit from having a strongly organized structure of their week with distinct events denoting temporal landmarks. Indeed, it has been shown that providing different scales of temporal information by changing the presentation format of calendar dates (monthly or weekly) is sufficient to change one’s temporal landmarks. This notably affected prospective thinking (Hennecke & Converse, 2017). Providing reliable temporal landmarks and routines to help better structure daily temporalities may provide a major framework for temporal orientation in various neuropsychological and neurological disorders.

Thus, contrary to our initial working hypothesis that the DoW effect would disappear or weaken during lockdown, the more robust effect we observed was instead a general slowing down of RTs. The slowdown during lockdown increased with the governmental stringency level and with the decrease in transit mobility. Whether this slowing down is selective to the DoW effect, to temporal cognition only, or can be generalized to all cognitive tasks recorded during lockdown is unclear. Using the same database, Chaumon et al. (2022) showed that transit mobility and stringency indices correlated with a slow-down of RTs in some temporal tasks, but not in others. For example, transit mobility and stringency did not correlate with RTs when participants were estimating the subjective distance to next week, but they did so when participants estimated how much time had elapsed since they had last logged on the website to do the tasks (retrospective duration). We cannot conclude that all aspects of temporal cognition were accompanied by a slowing down of chronometry. In past severe isolation experiments (Oléron et al., 1970), RTs were found to decelerate over the course of isolation, an effect the authors interpreted as a decrease in vigilance (Van Wassenhove, 2022). To some extent, our interpretation of this effect is milder: while the weekly cognitive map anchored by weekends subsisted in times of lockdown and partial isolation (namely, a resilience of the structure of memories), its full access may have been rendered more difficult to immediate recall and awareness of participants, in a time at which other means to synchronize were developed technologically, making virtual accessibility to others paradoxically easier. Thus, an important observation against the general interpretation that lockdowns felt as if days were blurring – hence, “Blursday” – our results show that the identification of days was not impaired during lockdowns, but that their access was. As historically observed in cognitive science, introspection is often a very bad descriptor of how the brain may compute information. With our study, we suggest that the felt Blursday phenomenon is not the outcome of our brains forgetting which day it is or forgetting to keep track of time, but likely more of a general sluggish cognitive access that is seen here in the retrieval of the names of the days but has also been reported in other tasks (Chaumon et al., 2022). In other words, our results speak to a more domain-general cognitive slow-down than a specific distortion of the temporal cognitive map.

Nevertheless, and quite surprisingly, the DoW effect appeared even more robust during lockdown: only then did we find that RTs for Thursday were significantly slower than for Monday, Tuesday, and Sunday. Graphic observation would suggest that this is due to a stronger slowing down for Thursday during lockdown than for any other day, though post hoc contrasts did not show a significant difference in the effect of lockdown between days. A tentative interpretation would be that the anchoring role of the weekend remains strong during lockdown, and preserves orientation to the end of the week, but the middle of the week becomes more difficult to access. Contrasting our original hypothesis, we can now put forward a new perspective: rather than blurring together, it may be that the mental distance between the middle of the week and the weekend has increased during lockdown. Further research would be needed to confirm this hypothesis, and test how it links to the phenomenology of time slowing down reported in many studies (Cellini et al., 2020; Chaumon et al., 2022; Cravo et al., 2022; Droit-Volet et al., 2020, 2021; Ogden, 2020, 2021; Van Wassenhove, 2022).

For instance, Rouhani et al. (2023) contrasted two possible hypotheses regarding remembered time during the pandemic. Their first hypothesis was that the pandemic state would disrupt the structural representation of events yielding a temporal dilation of events taking place during the pandemic period. Their second hypothesis was that the lack of contextual changes within the lockdown would reduce the number of memories and lead to a compressed estimation of events within this episode. This hypothesis followed from lab observations emphasizing the role of event boundaries on the temporal organization of memories (e.g., Clewett et al., 2019) in which novelty increases distances (i.e., temporal dilation). The authors found that pairs of events spanning the lockdown episode were judged as being closer to each other than pairs outside the lockdown episode, suggesting that fewer contextual changes led to temporal compression. This result provided support to their second hypothesis. The observation is also in agreement with the lengthening of temporal distances observed in the Blursday database (Chaumon et al., 2022), in which the lonelier a participant felt, the more distant the past or future was rated to be. It is plausible that we capture competing effects of these two hypotheses in our data: the trend towards a lengthening of distances between midweek days and weekends would suggest a stronger hierarchy in the weekly structure, though the overall slowing down of RTs during lockdown points to difficulties accessing this same structure. As such, while the weekend landmark may define stronger boundaries between episodes in the structuration of memory, the retrieval of the time of events may still be impaired. In sum, when the lack of novel events leads to a distortion of temporal distances, cyclical temporal structures may still provide robust landmarks to orient in time.

Altogether, our observations suggest that cultural landmarks may be resilient to contextual disorganization of social activities and can continue providing a cognitive anchor when orienting in time, structuring and stabilizing cognitive maps through changes. This speaks to the architecture of cognitive maps, which may be flexible and adaptive to change, but strongly grounded by landmarks such as cultural habits. The resilience of the DoW effect also highlights the importance of cyclic temporal landmarks, recurring at fixed frequency, and which allows structuring the linear flow of events, while introducing a metric to measure temporal distances.

### Limitations

Our study also has limitations. First, the task was conducted in the context of a much larger online study and we could not specifically counterbalance the number of samples across days and sessions. Though the indices of stringency and of transit mobility provided alternative ways for analyzing the data per session, and could potentially compensate for this, they could not be controlled and the number of samples varied across levels of stringency index and transit mobility index. The lack of a balanced design may have prevented capturing the hypothesized interactions between the day of the week and the different measures of lockdown. In any case, as it is, we cannot conclude on a differential effect of lockdown for each day of the week and weekend. Another limitation concerns the definition of the stringency of government measures and control conditions. We could not fully control for the regional diversity in stringency. In France, different regions may have had different sanitary measures, but we did not have access to the participants’ region. The control session was conducted after the lift of lockdowns, but the living conditions and social habits were not fully back to the pre-lockdown conditions. As such, the stringency and mobility indices were helpful in providing a more refined scale to relate the different sessions to the pre-lockdown state. Moreover, fully returning to pre-lockdown conditions might not be possible in the near future: moderate sanitary measures are still being applied and the pandemic episode remains a salient event in memory.

## Conclusion

In an online study conducted on the French population during the COVID-19 pandemic, participants were asked to answer the question “What day is today?” as fast as possible. Participants were faster in correctly identifying a day during a weekend as compared to a weekday, consistent with the DoW effect. This effect was resilient during the lockdown. However, RTs were slower during lockdown, and increasingly slower with increased stringency and lack of mobility. This observation is consistent with previous studies implying that the disruption of social habits during the crisis was associated with general time distortions. The observation that weekends maintained a facilitating effect on RTs during lockdown suggests that culturally grounded temporal cognitive maps may provide an important anchoring role in orientation to time at the individual, population, and social scale.

## Supplementary information

Below is the link to the electronic supplementary material.

Supplementary file1 (DOCX 1401 KB)

## Data Availability

All data are publicly available as part of the Blursday database (https://dnacombo.shinyapps.io/Blursday/).

## Code availability

All analyses were conducted using R (v4.1.1; R Core Team, 2021) with packages tidyverse (v1.3.1; Wickham et al., 2019), lmerTest (v3.1.3; Kuznetsova et al., 2017), effects (v4.2.0; Fox, 2003; Fox & Weisberg, 2019), emmeans (v1.7.2; Lenth, 2022), and car (v3.0.4; Fox & Weisberg, 2019). Analyses scripts are available as an R notebook on the Open Science Framework (https://osf.io/cem3h/).

## Declarations

### Open practices statement

The data and materials are available at the links provided. This experiment was not preregistered.
