# Supplementary material for: The day-of-the-week effect is resilient to routine change
Source: Mem Cognit. 2024 Jul 16;53(3):792–803. doi: 10.3758/s13421-024-01606-8 (PMC12052915; doi:10.3758/s13421-024-01606-8)
Supplement: Supplementary file 1 — Supplementary file1 (DOCX 1401 KB) [file 13421_2024_1606_MOESM1_ESM.docx]

Supplementary materials

As Supplementary Materials, we provide further description of the pattern of reaction times (RTs) across sessions (Fig. S1), and of the outlier exclusion criteria (Fig. S2). We also explore whether successive answers to the question “What day of the week is it?” within one day modulated the day of the week (DoW) effect. Furthermore, we provide a descriptive analysis of work habits during and outside of lockdown, and how they may have interacted with the DoW effect (Fig. S3-8)

**Work habits inside and outside of lockdown**

As part of the Blursday database (Chaumon et al., 2022), the work habits of participants in and out of lockdown were collected. Considering only clean data points (see Statistical Analysis), this provides 189 data points in S1 (from 139 participants), 136 data points in S2 (from 87 participants), 148 data points in S3 (from 72 participants), 90 data points in S4 (from 90 participants) and 246 data points in SC (from 165 participants).

We aim to answer four questions:

(1) If indeed the day of the week effect is induced by the typical weekly work cycle, then differences in work habits should be a driving factor on the impact of lockdown on the day of the week effect. Therefore, we expected that participants who worked remotely during lockdown, but worked outside their home once the lockdown was over, would exhibit the greatest difference in behavioral patterns. On the other hand, participants who kept working at home, even when the lockdown was over, would show a smaller difference in their day of the week effect. Thus, was the impact of lockdown on the day of the week effect greater for participants working outside once the lockdown was over, compared to participants who kept working at home?

(2) Similarly, we hypothesized that participants who were most affected by the lockdown were those driven to change their work habits during this period. Was the impact of lockdown greater for participants who used to work outside, but had to work at home during lockdown, compared to participants who did not change their habits?

(3) On the other hand, participants with unusual work habits, independent of the common weekly cycle, could show a weaker day of the week effect. Does having work habits that are independent of the usual weekly cycle, such as working at night, also disrupt the day of the week effect?

(4) If the hypotheses above are proven to be correct, we would need to assess whether the measures of social disruption we used also reflect changes in work habits. Do work habits align with measures of stringency and mobility?

Due to the lower number of data points, we do not have the statistical power necessary to properly test each of these hypotheses. However, we report a comprehensive descriptive analysis for each of these questions.

***Was the impact of lockdown on the day of the week effect greater for participants working outside once the lockdown was over, compared to participants who kept working at home?***

Participants reported whether they were working from home or from outside of home over the current week, on a scale of 0 (“work mostly at home”) to 100 (“work mostly outside of home”). For visual inspection, we divided this scale into three groups, participants who mostly worked at home (0-33), participants who reported working either at home or outside (34-66) and participants who mostly worked outside (67-100).

As expected, during S1 participants overwhelmingly reported working at home. However, we may note that in SC, the proportion of participants working at home and working outside was more balanced.

If work habits are a driving factor of the day of the week effect, we expect a strong contrast between participants who worked at home during lockdown, and participants who worked outside once the lockdown was over. On the other hand, we would expect less difference between participants who worked at home, whether in lockdown or not. Therefore, we compared participants who worked at home during S1 and participants who worked outside or at home during SC.

Whether working at home or not, in or out of lockdown, participants show a peak of RTs in the middle of the week. However, it seems that this peak is on Wednesday when working at home, whether in or out of lockdown; and on Thursday when working outside when out of lockdown. Though participants working at home in S1 and in SC exhibit a similar pattern of RTs, participants in SC seem much faster, especially in the middle of the week. However, we do not have enough data points on Monday, Tuesday and Sunday in SC to conclude further on a difference in the day of the week effect.

Participants also reported how their working habits changed once the lockdown was over (SC), compared to when they were in lockdown (S1), on a scale of 0 (“work more at home”) to 100 (“work more outside of home”). For visual inspection, we divided this scale into three groups, participants who mostly worked more at home (0-33), participants whose working habits did not change a lot (34-66) and participants who worked more outside (67-100). Only 40 participants reported no or little change, and the data points are not sufficient to cover all days of the week. Therefore, we only show the pattern of RTs for participants who worked more at home, by contrast to those who worked more outside.

Overall, no matter the changes in work habits, participants seem to show a peak of RTs in the middle of the week. Once again, working at home seems to be associated with a peak on Wednesday, whereas working outside would be associated with a peak on Thursday.

***Was the impact of lockdown greater for participants who used to work outside, but had to work at home during lockdown, compared to participants who did not change their habits?***

In a similar way, participants reported how their working habits changed during the lockdown (S1), compared to three months prior, on a scale of 0 (“work more at home”) to 100 (“work more outside of home”). For visual inspection, we divided this scale into three groups, participants who mostly worked more at home (0-33), participants whose working habits did not change a lot (34-65) and participants who worked more outside (66-100). Once again, only 40 participants reported no or little change, and the data points are not sufficient to cover all days of the week. Therefore, we only show the pattern of RTs for participants who worked more at home, by contrast to those who worked more outside.

Most participants worked more at home during the lockdown than in prior months (129 over 189 data points, see also Fig. S5). The overall day of the week pattern observed during lockdown seems to be robust even for participants who were the most affected by the lockdown.

***Does having work habits that are different from the usual weekly cycle, such as working at night, also disrupt the day of the week effect?***

In another questionnaire of the Blursday database, participants reported whether they were working at night on the previous day. Therefore, we have partial data on participants’ night work habits.

Night workers seem to show a peak of RTs around Wednesday rather than Thursday. Overall, the DoW effect seems less clear for night workers. However, the lack of data points does not allow us to draw strong conclusions.

***Do work habits align with measures of stringency and mobility?***

Visual inspection of the data suggested that different work habits may shift by a day the pattern of the day of the week effect, though it remained robust across conditions. Furthermore, the global slowing down of RTs induced by lockdown was consistent for both remote workers and outside workers, though less clear for night workers. Therefore, the effects we describe in this study remain stable despite some differences in work habits across participants. Overall, work habits were quite homogeneous: remote work was almost ubiquitous during the lockdown (85.83 % of responses in S1, Fig. S3), and night workers were very few in all sessions (7.39% of responses across sessions, Table S1). This prevents us from doing proper statistical tests to assess the actual effect of work habits, but consolidates our conclusions. In order to assess whether the measures of social disruption that we used aligned with work habits, we inspected the relation of remote working with stringency and transit mobility indices.

Reports of remote working tended to align with increasing stringency, and decreasing mobility. This confirms that these two measures captured the overall situation of the population across sessions.

**Supplementary Figure 1.**

*RTs over days of the week.*

**
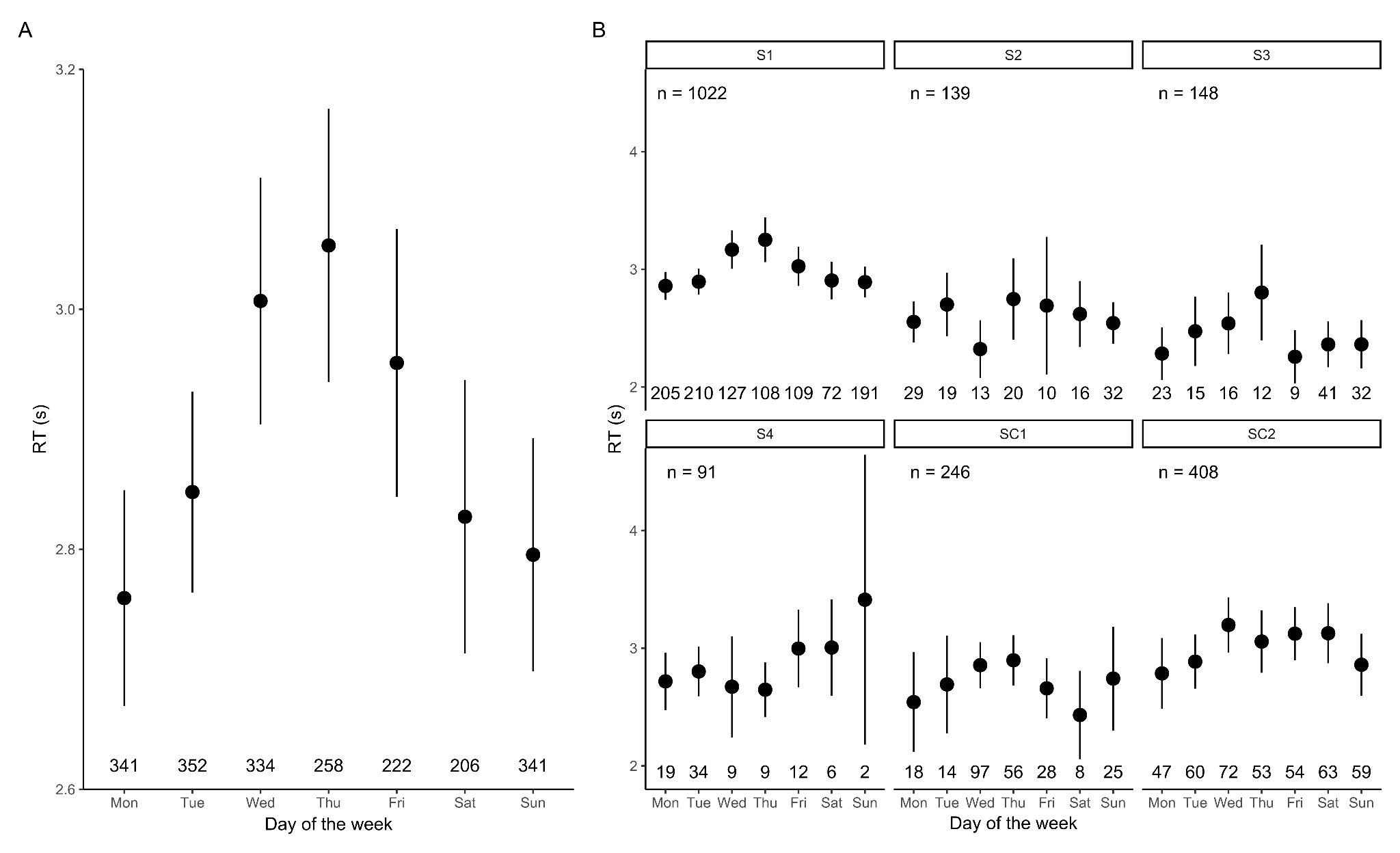
**

(A) RTs over days of the week, all sessions (S1-S4 and SC) pooled together. Points are mean estimates. Error bars are two standard errors of the mean (s.e.m). The total number of data points is given in the top left corner, and number of data points for each day of the week are given at the bottom. (B) RTs over days of the week, for each session separately. The two datasets collected for the control session are shown separately (SC1, first control data collection in 2021; SC2, second control data collection in 2023; see Fig. 1).

**Supplementary Figure 2.**

*RTs over days of the week, in S2, S3 and SC, with (orange) and without (green) data points collected in August and July.*

**
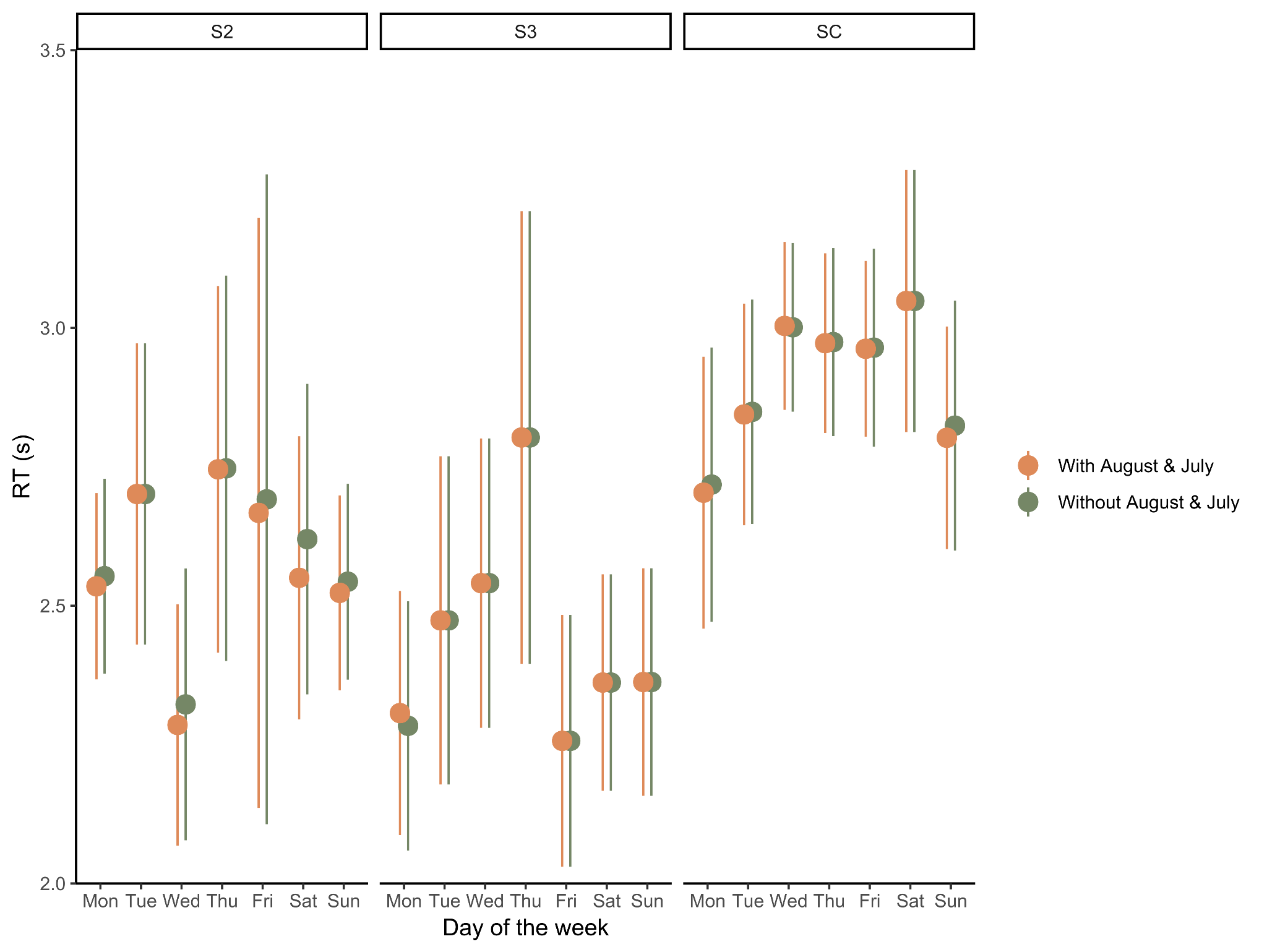
**

Points are mean estimates, error bars are two s.e.m.. Visual inspection suggests that the exclusion of data points in August and July (58 data points) does not change the DoW effect.

**Supplementary Figure 3.**

*RTs over days of the week for first and non-first responses.*


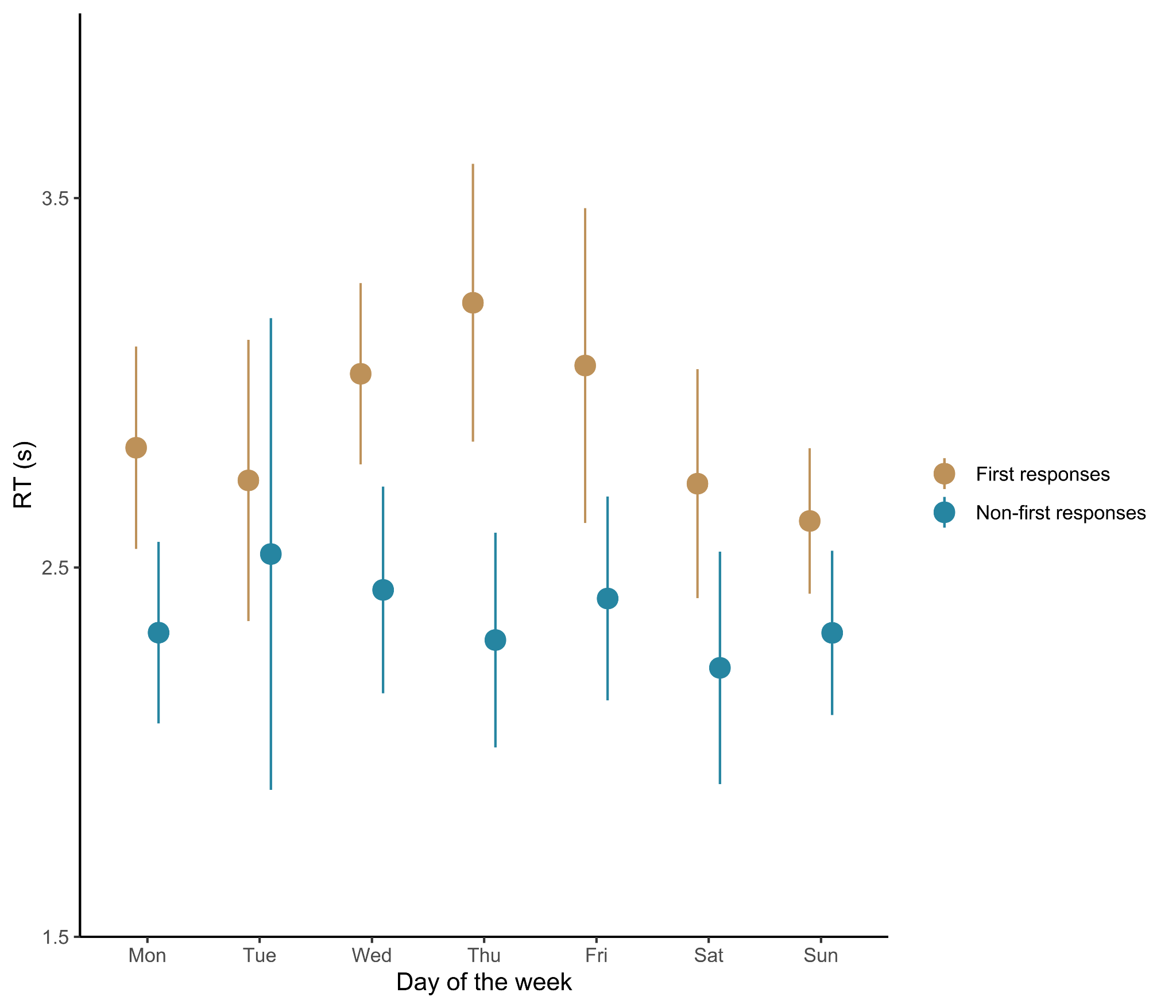


Considering only clean data points (see Statistical Analysis), 115 participants answered multiple times within one day across sessions (S1, S2, S3 and SC; no participants in S4). Selecting only responses from these participants on that same day allowed contrasting 139 non-first responses with 174 first responses. As non-first responses represent only a small proportion of the data (6.77% of responses across all sessions), we could not properly test for a possible interaction with the DoW effect. Points are mean estimates. Error bars are two s.e.m.

**Supplementary Figure 4.**

*Trend of RTs over days of the week, in S1, S2, S3 and SC, with (blue) non-first responses and without (brown) non-first responses.*


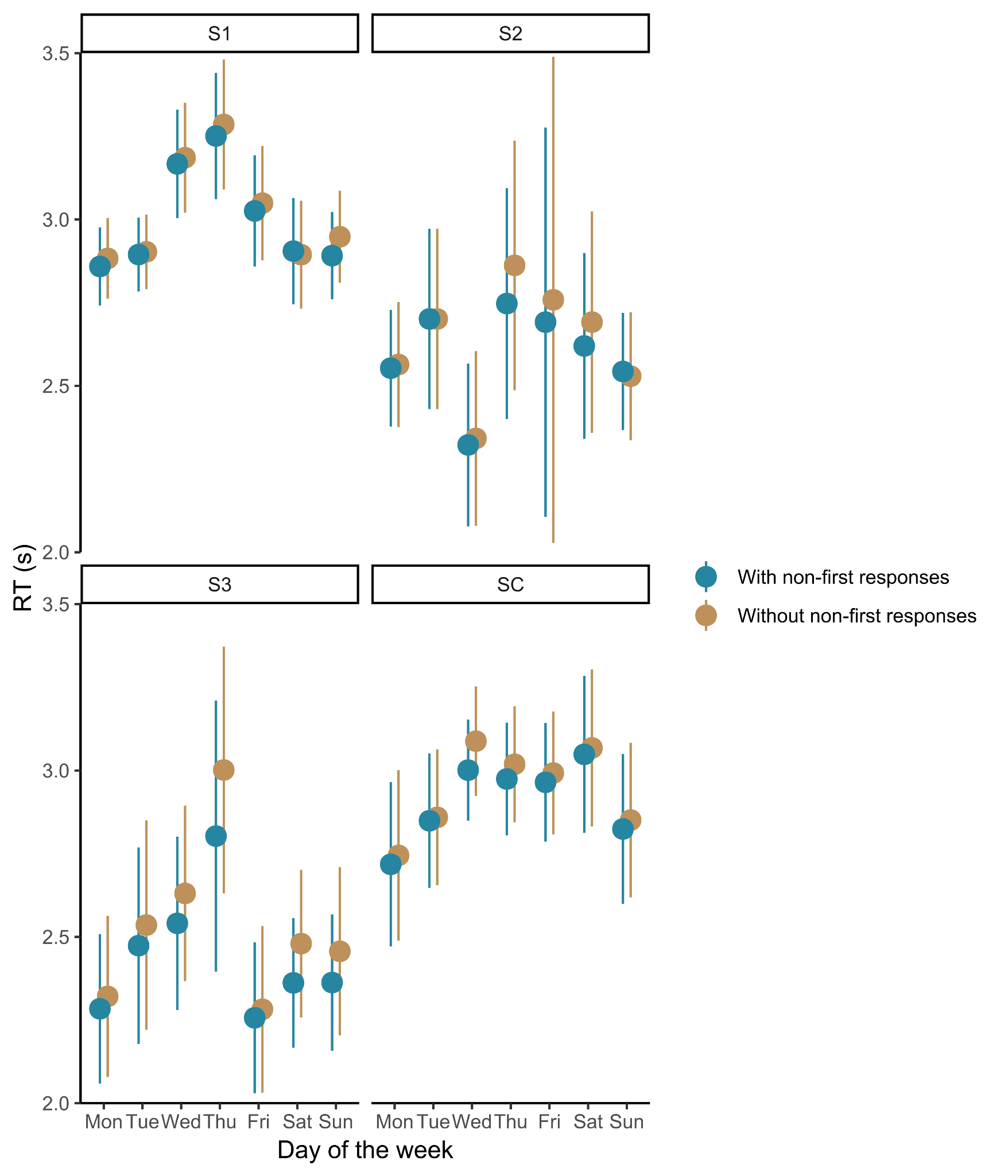


Points are mean estimates. Error bars are two s.e.m.

**Supplementary Figure 5.**

*Proportion of participants who worked mostly at home (red), either at home or outside (yellow), or mostly outside of their home (green), across sessions.*

**
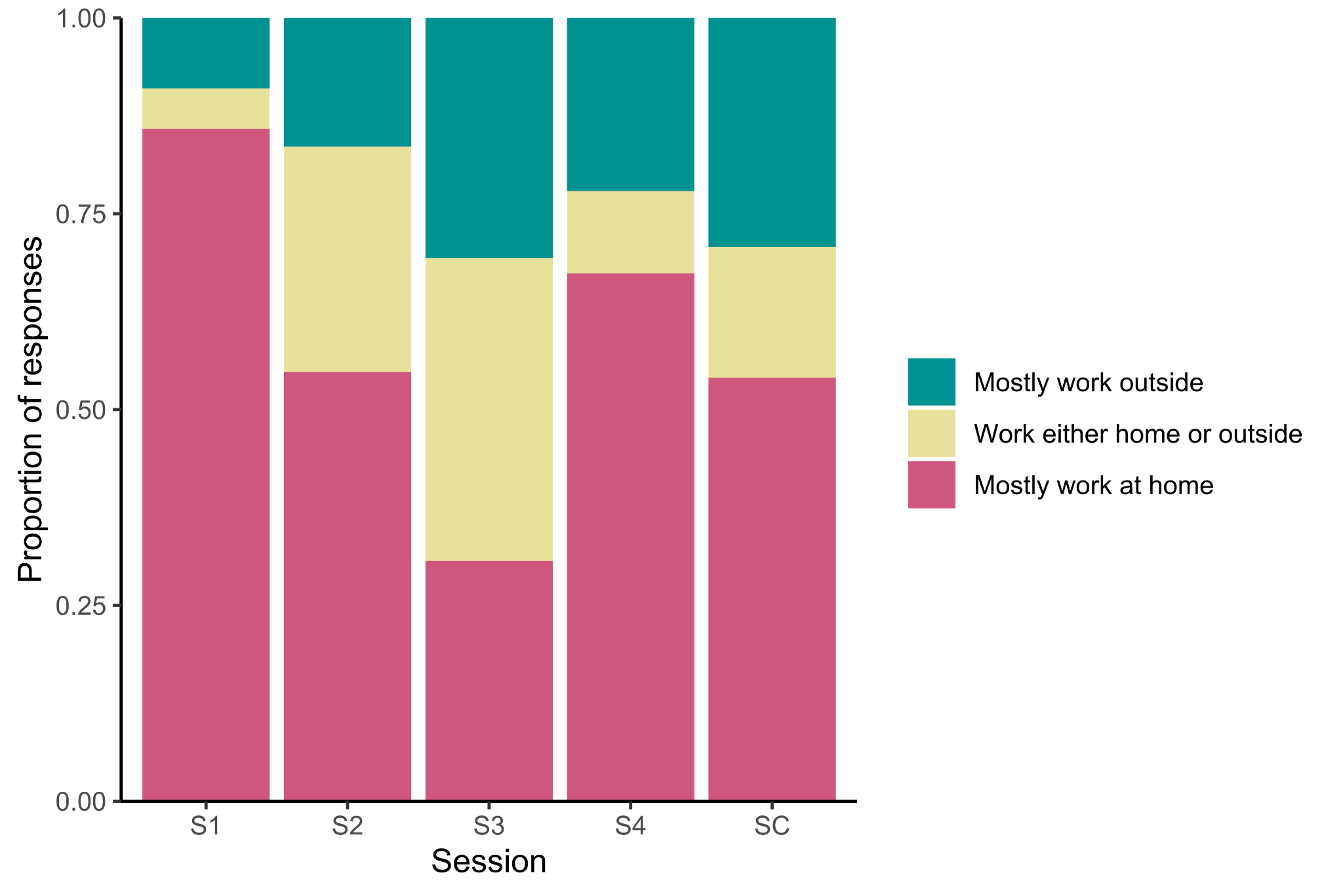
**

**Supplementary Figure 6.**

*RTs as a function of the day of the week, for participants working at home during the lockdown (S1) and participants working at home or outside, during the control session (SC).*

**
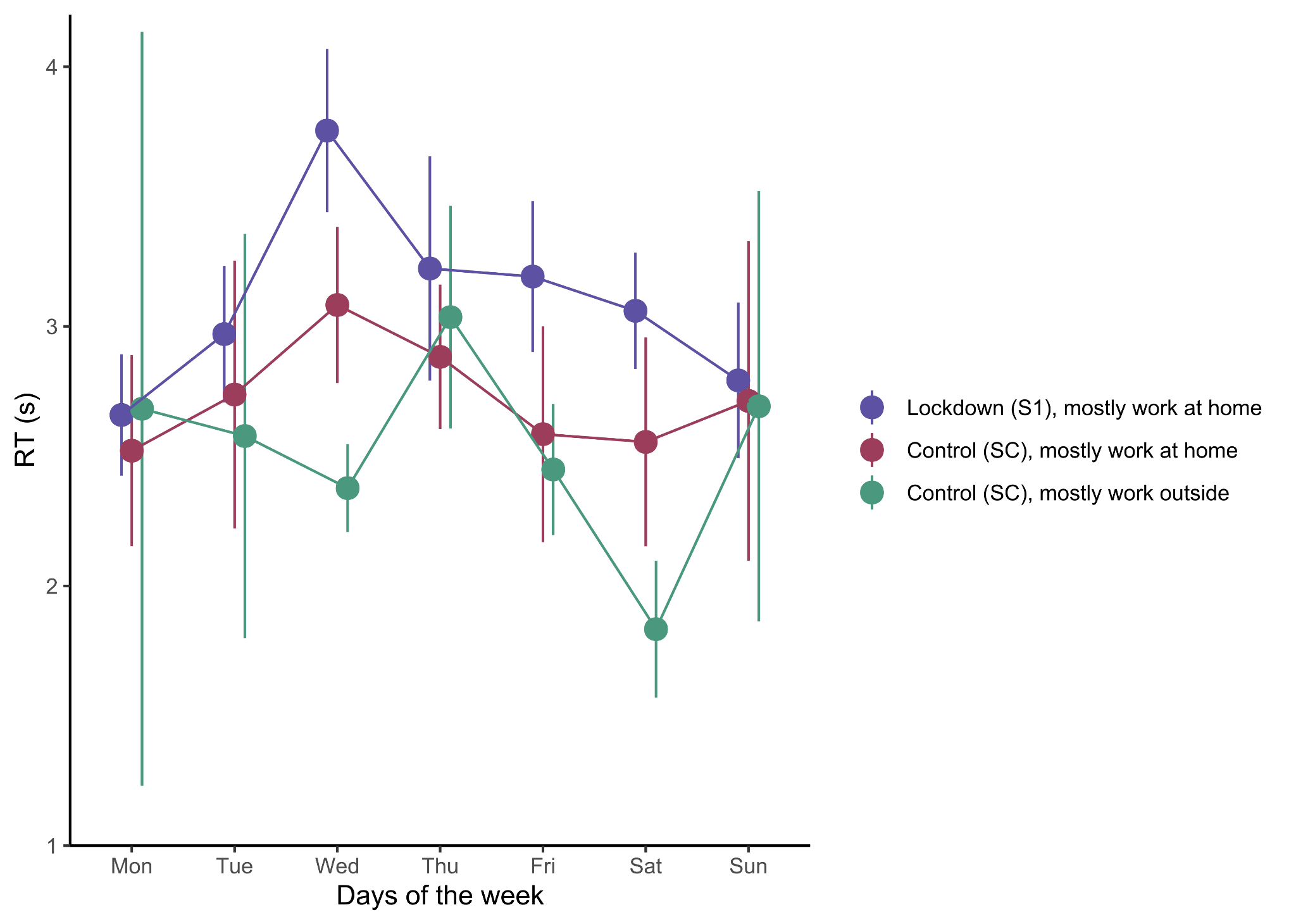
**

Dots are mean estimates. Bars are two s.e.m.

**Supplementary Figure 7.**

*RTs as a function of the day of the week, for participants who worked more outside in SC compared to S1, and participants who worked more inside in SC compared to S1.*

***
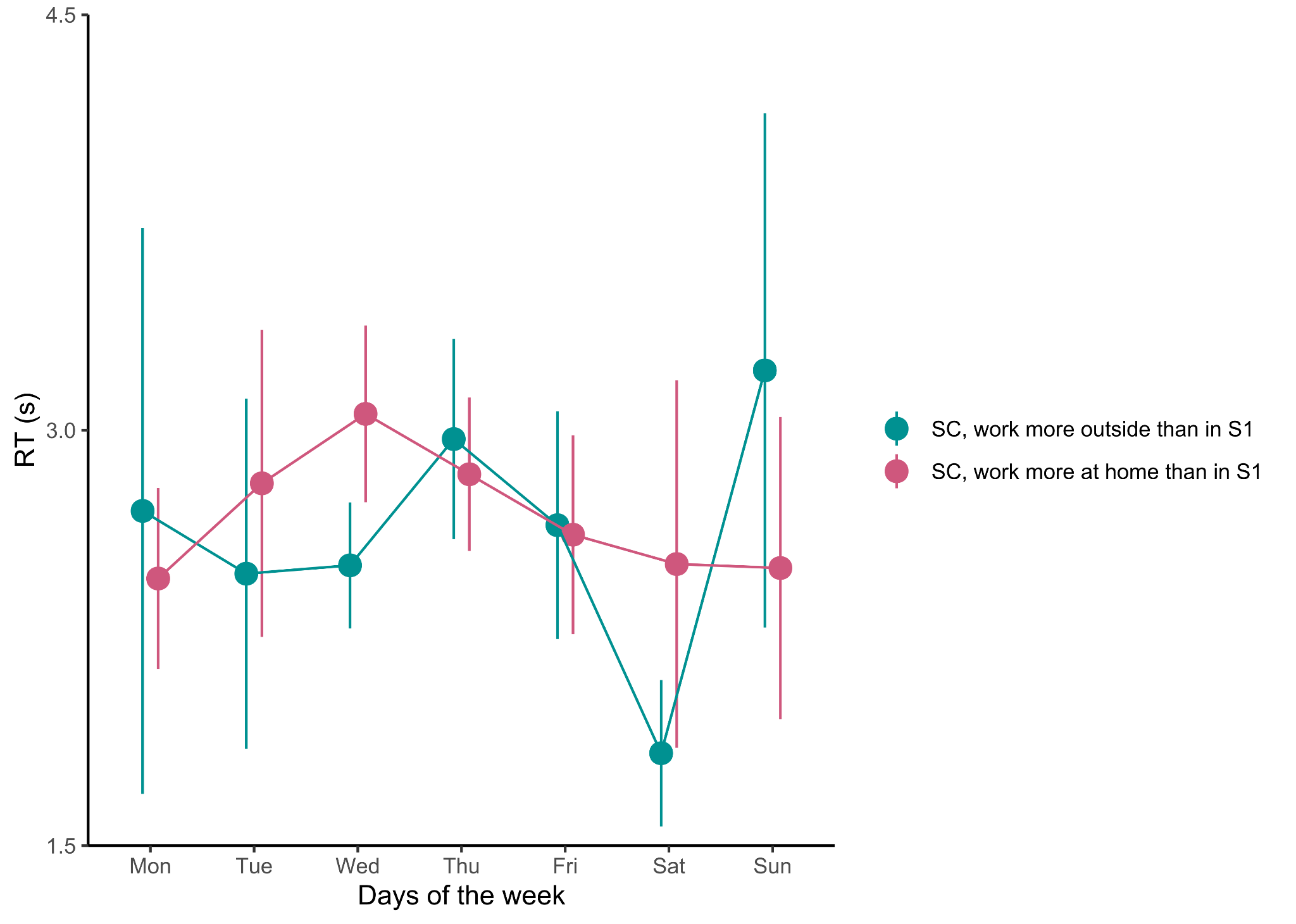
***

Dots are mean estimates. Bars are two s.e.m.

**Supplementary Figure 8.**

*RTs as a function of the day of the week, for participants who worked more often outside in S1 compared to three months prior, and participants who worked more often inside in S1 compared to three months prior.*


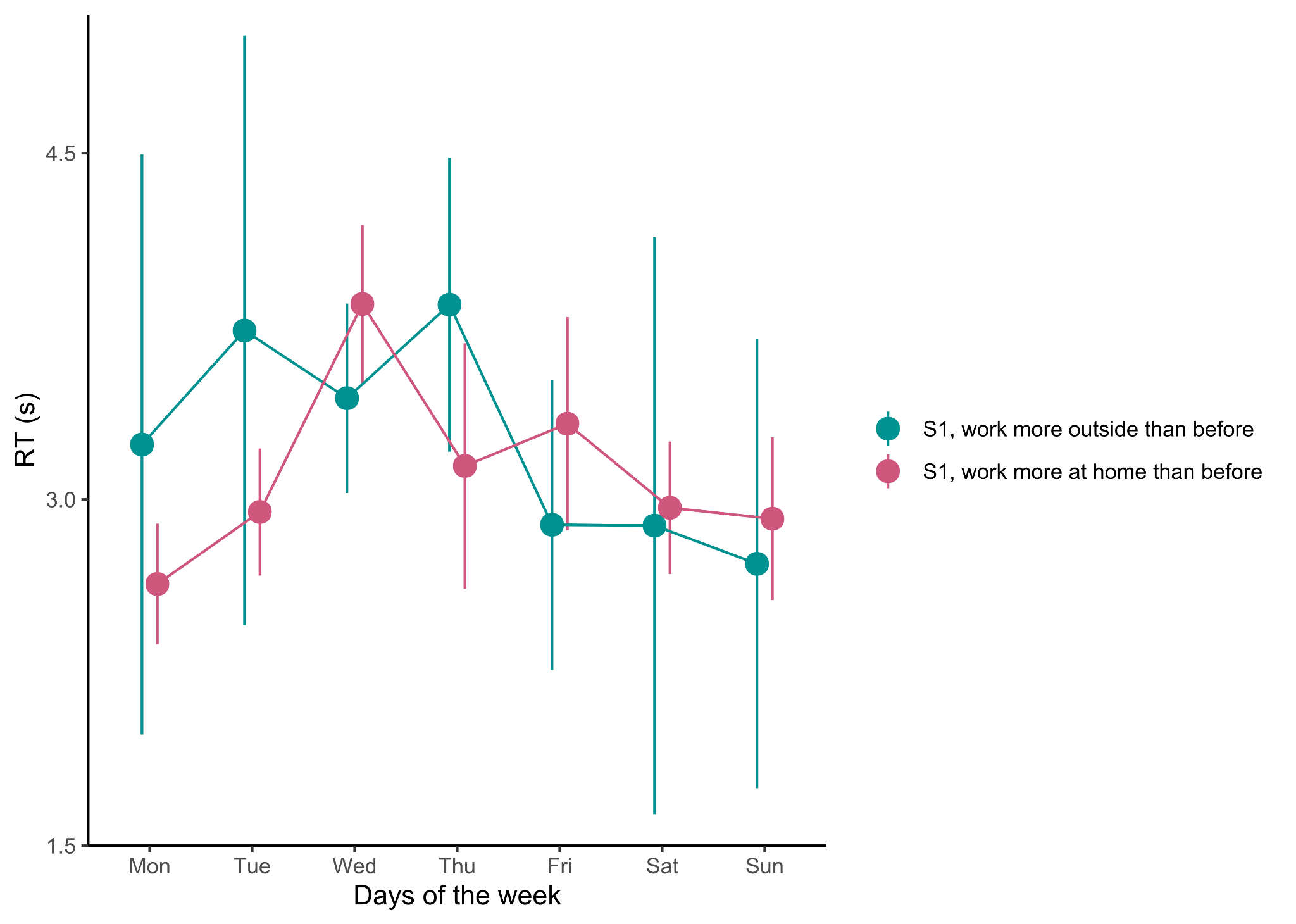


Dots are mean estimates. Bars are two s.e.m..

**Supplementary table 1.**

*Number of responses and of participants reporting working the previous night or not.*

| Session | Worked at night | Did not work at night |
| --- | --- | --- |
| S1 | 76 data points (70 participants) | 904 data points (737 participants) |
| S2 | 10 data points (10 participants) | 117 data points (78 participants) |
| S3 | 6 data points (5 participants) | 122 data points (69 participants) |
| S4 | 22 data points (22 participants) | 68 data points (68 participants) |
| SC | 33 data points (26 participants) | 210 data points (144 participants) |

As above, we only consider clean data points (see Statistical Analysis).

**Supplementary Figure 9.**

*RTs as a function of the day of the week, for participants who worked the previous night or not, during lockdown (S1) and outside of lockdown (SC).*

**
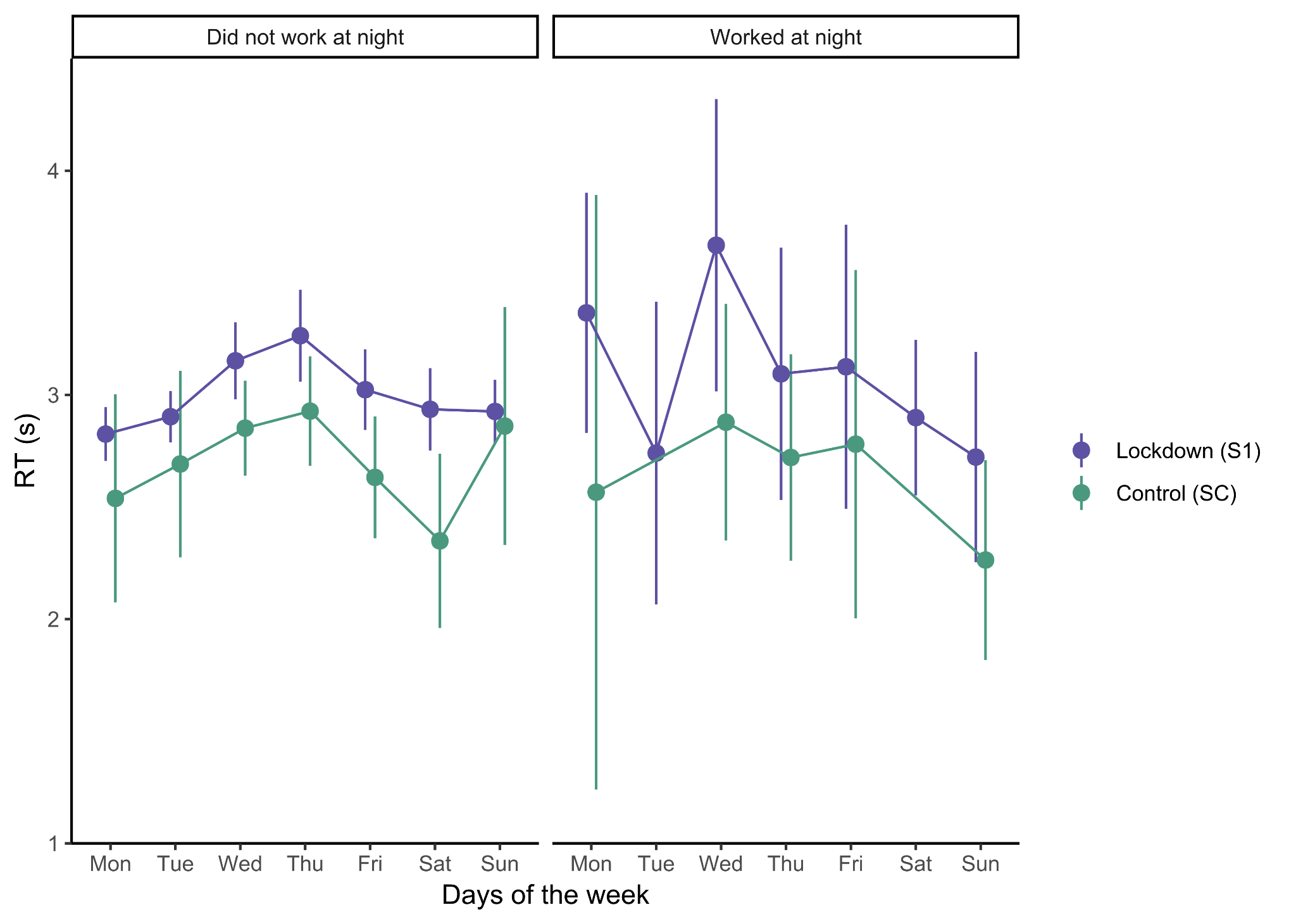
**

Dots are mean estimates. Bars are two s.e.m..

**Supplementary Figure 10.**

*Reported work habits aligned with measures of government responses.*

**
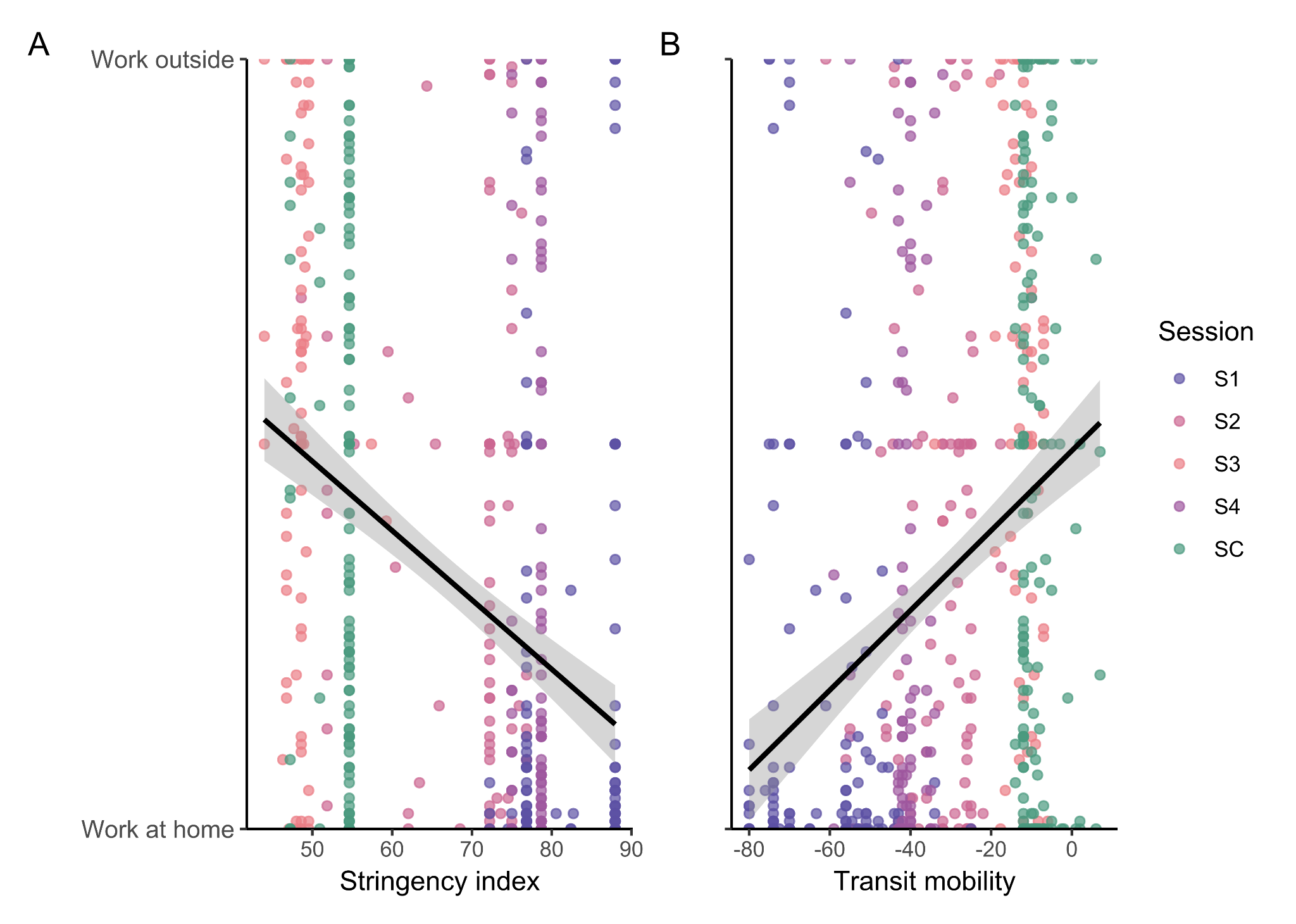
**

(A) Work habits as a function of the stringency index. Each point is a participant. When several data points were available for one participant in one session, the stringency index was averaged across these points. Work habits range on a continuous scale from “mostly work at home” (0) to “mostly work outside” (100). The black line is a simple linear regression of work habits over stringency, the gray ribbon is the 95% confidence interval. Sessions are represented in color. (B) Work habits as a function of the transit mobility index. Each point is a participant. When several data points were available for one participant in one session, the transit mobility index was averaged across these points. The black line is a simple linear regression of work habits over transit mobility, the gray ribbon is the 95% confidence interval.

**Supplementary table 2.**

*Demographic summary, by session.*

| Session | Number of data points | Number of participants | Sex | | Mean age | Handedness | | | Remote Working | | | Nightwork |
| --- | --- | --- | --- | --- | --- | --- | --- | --- | --- | --- | --- | --- |
|  |  |  | Men | Women |  | Right-  handed | Left-handed | Ambidextrous | Worked mostly at home | Worked either home or outside | Worked mostly outside |  |
| S1 | 1022 | 812 | 206 | 531 | 45.1 (s.d. = 15) | 648 | 68 | 21 | 118 (84.9%) | 11 (7.91%) | 10 (7.19%) | 76 (7.76%) |
| S2 | 139 | 88 | 20 | 66 | 50.2 (s.d. = 14.5) | 74 | 7 | 5 | 49 (56.3%) | 24 (27.6%) | 14 (16.1%) | 10 (7.87% |
| S3 | 148 | 72 | 13 | 56 | 51.1  (s.d. = 15.2) | 58 | 7 | 4 | 21 (29.2%) | 26 (36.1%) | 25 (34.7%) | 6 (4.69%) |
| S4 | 90 | 90 | 11 | 23 | 50.6  (s.d. = 16) | 27 | 4 | 3 | 64 (71.1%) | 9  (10%) | 17 (18.9%) | 22 (24.2%) |
| SC1 | 246 | 165 | 39 | 122 | 31.2  (s.d. = 12.7) | 139 | 18 | 4 | 84 (50.9%) | 28  (17%) | 53 (32.1%) | 33 (13.6%) |
| SC2 | 408 | 408 | 190 | 216 | 29.4  (s.d. = 10.1) | 28 | 1 | 1 | *NA* | *NA* | *NA* | *NA* |

We report the number of data points and the number of participants, with the sex, age and handedness distribution of the clean data set that was effectively used in our analyses (see Statistical Analysis). Race and ethnicity were not collected. Note that demographic data is missing for some participants. In particular, handedness was only collected for a few participants in SC2. Additionally, we report work habits for each session. Note that this demographic data was not collected for SC2. As described above, remote working was discretized into three groups: participants who worked mostly at home, participants who worked mostly outside, and participants who worked either at home or outside. We report the number of participants for each group, as well as the proportion this group represents within the session. Finally, we report the number of participants who had worked the night before, as well as the proportion that they represent within the session. As work habits were not collected for all participants, the proportions are calculated based on available data points only.
